# Fabrication of nanoemulsion delivery system with high bioaccessibility of carotenoids from *Lycium barbarum* by spontaneous emulsification

**DOI:** 10.1002/fsn3.2863

**Published:** 2022-04-20

**Authors:** Chunlan Zhang, Bin Li

**Affiliations:** ^1^ College of Life Sciences Tarim University Alar China; ^2^ College of Food Science and Technology Huazhong Agriculture University Wuhan China; ^3^ Production & Construction Group Key Laboratory of Special Agricultural Products Further Processing in Southern Xinjiang Alar China; ^4^ Key Laboratory of Environment Correlative Dietology (Huazhong Agricultural University) Ministry of Education China

**Keywords:** bioaccessibility, carotenoids, nanoemulsions, spontaneous emulsification

## Abstract

The interest in incorporating carotenoids into foods and beverages is growing due to their potential health benefits. However, the poor water solubility and low bioavailability of carotenoids are still challenges in food application. This work aimed to study the influence of system composition and preparation conditions on the physical properties of carotenoids‐loaded nanoemulsions prepared by spontaneous emulsification. Furthermore, the bioaccessibility of carotenoids in the nanoemulsions was evaluated. The nanoemulsions with the smallest droplet size were produced when the ratio of Span 80:Tween 80 was 1.5:8.5. The droplet size increased slightly with the increase of organic phase content (24%–40%). The droplet size decreased gradually with the increase of stirring speed (200–1000 rpm (revolutions per minute)). The ratio of mixed surfactants and surfactant‐to‐oil ratio (SOR) had an appreciable impact on the droplet size. Carotenoids‐loaded nanoemulsions with small mean droplet size (d < 50 nm) could be prepared with the optimized conditions. The initial digestion rate decreased as the SOR increased. The bioaccessibility could reach up to about 80% at SOR=2–5 in vitro digestion. These results have important implications for the design of effective delivery systems to encapsulate carotenoids and other lipophilic bioactive components in food applications.

## Graphical abstract

1. Carotenoids‐loaded nanoemulsions with small mean droplet size (d < 50 nm) could be prepared after the optimization of preparation conditions.
2. The high bioaccessibility could reach up to about 80% at SOR=2–5 in vitro digestion.

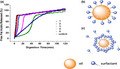

## 1 INTRODUCTION

Carotenoids are widespread existing in various fruits and vegetables, as well as some animal products. *Lycium barbarum*, a popular traditional Chinese herbal medicine, is well known in Asia for nourishing the liver and improving eyesight. The zeaxanthin and carotene in *Lycium barbarum* are recognized as the main bioactive compounds for improving eyesight (Luo et al., 2004). In addition, they may possess antioxidant, antitumor activities and enhance immunity due to the presence of various functional components (Gan et al., 2004; Qian et al., 2004). Zeaxanthin dipalmitate, a kind of predominant carotenoid in *Lycium barbarum*, is a focus in current research for its prevention of age‐related macular degeneration (Inbaraj et al., 2008; Peng et al., 2005; Zhou et al., 1999). Due to their potential health benefits, incorporation of carotenoids into functional foods or dietary supplements has aroused extensive interest in recent decades, especially for consumers and food manufacturers. However, there are still challenges in their poor water solubility and low bioavailability.

To overcome the limitations, a useful strategy is to encapsulate carotenoids into appropriate delivery vehicles. Various delivery systems have been developed to load lipophilic functional agents, including micelle, emulsions, colloids, biopolymer matrices, powders, and so on (McClements, 2013). Emulsion‐based delivery systems are available to deliver bioactive lipids for the large interest in the development of nutraceutical or functional foods. Emulsions, nanoemulsions, and microemulsions are distinguished by their particle dimensions and thermodynamic stability (Mcclements, 2011). Differences in particle dimensions lead to their different functional performances. The nanoemulsions with smaller droplets tend to have a higher stability to gravitational separation, flocculation, and coalescence than conventional emulsions. The droplets of nanoemulsions are so small that they only scatter light waves weakly. Therefore, they can be incorporated into optically transparent products without adversely affecting their clarity. Nanoemulsion‐based delivery system can not only increase the solubility and stability, but also improve the bioavailability of lipophilic agents, which is considered an important target for nutrients delivery system (Huang et al., 2010).

The pH‐stat method in vitro digestion has been widely used in food and pharmaceutical research to study the characterization of lipid digestion and bioaccessibility of active components (Yan et al., 2011). Previous studies have shown that the rate and extent of lipid digestion depended on numerous factors, including droplet size (Lee et al., 2011; Salvia‐Trujillo et al., 2013; Troncoso et al., 2012; Yi et al., 2014), oil type (Lee et al., 2011; Salvia‐Trujillo et al., 2013; Troncoso et al., 2012; Yi et al., 2014), lipid physical state (Bonnaire et al., 2008), and interfacial composition (Bonnaire et al., 2008). At the same time, the emulsions droplets could interact with various surface‐active components, so the lipid digestion was also influenced by bile salts, pepsin, lipase, and minerals (Ca^2+^and Na^+^). Previous results showed that emulsifier affected the absorption of pancreatic lipase onto the surface of emulsified fats (Mun et al., 2007). The effects of surfactants with low‐molecular weight on the digestibility of lipids were further studied by Li. They found that the different types of surfactants have different inhibitions on lipid digestion (Yan et al., ). Low‐molecular weight nonionic surfactants are the commonly used emulsifiers for the fabrication of nanoemulsions by spontaneous emulsification. However, the effects of low‐molecular emulsifier content and mixing ratio on carotenoids‐loaded emulsions droplet size, lipid digestion, and bioaccessibility have not been thoroughly studied.

In the paper, nanoemulsion systems were fabricated to encapsulate carotenoids from *Lycium barbarum* by spontaneous emulsification, aiming to improve the hydrophilicity and bioaccessibility of carotenoids. The influences of mixed surfactants ratio (Span 80:Tween 80), surfactants‐to‐oil ratio (SOR), organic phase content, and stirring speed on the physical properties of carotenoids‐loaded nanoemulsions were studied. Furthermore, the influence of SOR on the lipid digestion and bioaccessibility of carotenoids in the nanoemulsions was investigated using the pH‐stat method. The current study would have important implications for the design of effective delivery systems to encapsulate carotenoids and other lipophilic bioactive components in food or pharmaceutical applications.

## 2 MATERIALS AND METHODS

### 2.1 Materials

*Lycium barbarum* pigment oleoresin (20% by mass carotenoids) was prepared by the Center for Biomacromolecules Research, Huazhong Agricultural University. Medium‐chain triglyceride (MCT) was obtained from Boxing Chemical Reagent Co. Ltd. Pancreatic lipase and porcine bile extracts were purchased from Sigma‐Aldrich. Tween 80, Span 80, sodium azide, ethanol, and n‐hexane were purchased from Sinopharm Chemical Reagent Co. Ltd. All other chemicals used were of analytical grade, unless otherwise stated. Double‐distilled water was used for the preparation of all solutions.

### 2.2 Fabrication of carotenoids emulsions

Emulsions were prepared by spontaneous emulsification using the method described previously with some modifications (Saberi et al., 2013). First, the surfactants (Tween 80 and Span 80) were mixed with MCT containing 0.2 wt.% carotenoids. The mixtures were taken as organic phase. Then, the organic phase was added dropwise into aqueous phase (distilled water containing 0.02 wt.% sodium azide) under magnetic stirring for 15 min at room temperature. In the preparation, the factors of Span 80‐to‐Tween 80 mass ratio, organic phase content, stirring speed, and surfactants‐to‐oil mass ratio were investigated.

### 2.3 Droplet size and ζ‐potential measurements

The average droplet size of the emulsions was determined by a Nano‐ZS (Malvern Instruments). First, emulsions were diluted 100 times using 10 mM phosphate buffer solutions at pH 7.0 (PBS). Z‐average diameters for droplet size, polydispersity index (PDI), and ζ‐potential were measured. The particle sizes of the digesta were also calculated using the surface‐weighted mean diameters (d_3,2_) by Mastersizer 2000 for its big dimension.

### 2.4 Transparency measurements

The transparency of emulsions was measured at 600 nm using a spectrophotometer (UV‐1100, meipuda instrument, China). Prior to the measurements, double‐distilled water was used as a reference.

### 2.5 In vitro digestion

The in vitro digestion was carried out by simulated small intestinal conditions, as described previously (Yan et al., ). The pH‐stat (907 Titrando, Metrohm USA Inc.) was used to monitor and control the pH (pH 7.0) of the digestion process. Thirty milliliters of emulsions containing 0.5% MCT was placed into a bottle and then incubated in a water bath (37°C) for 10 min. Four milliliters of bile extract solution, containing 187.5 mg of bile extract (pH 7.0, PBS), was added into the 30 ml emulsions with stirring. Then, 1.0 ml of CaCl_2_ solution and 1.0 ml of NaCl were added into the bottle with final concentrations of 10 and 150 mM, respectively. The pH value of the system was adjusted to 7.0 with NaOH solution. After 1.5 ml of freshly prepared pancreatin suspension (40 mg/ml, pH 7.0, PBS) was added, the automatic titration experiment was started immediately. Digestion experiments were performed for 120 min at 37°C. The amount of free fatty acids (FFAs) released during the digestion process was calculated from the following equation.

$$\text{FFA released}\left(\%\right)=100\times \left(C_{\text{NaOH}}\times V_{\text{NaOH}}\times M_{\text{oil}}\right)/\left(2\times m_{\text{oil}}\right).$$

where *C*_NaOH_ is the molarity of the NaOH solution used to titrate the sample (mol/L), *V*_NaOH_ is the volume of NaOH solution required to neutralize the FFAs produced at digestion, and *M*_oil_ is the molecular weight of the oil (g/mol). The molecular weight of the MCT was taken to be 500 g/mol, and *m*_oil_ is the total mass of oil initially present in the incubation cell (g).

### 2.6 Bioaccessibility determination

The bioaccessibility of carotenoids‐loaded nanoemulsions was determined by the in vitro digestion process using a method described previously (Qian et al., 2012). An aliquot of the raw digesta was centrifuged at 16,000 rpm for 30 min at 4°C. The middle transparent layer was taken to be the ‘‘micelle fraction’’ in which the carotenoids were solubilized. Two milliliters of the micelle fraction was mixed with equivalent mixed solvent (n‐hexane:ethanol = 1:1), vortexed, and centrifuged at 4000 rpm for 10 min at 25°C. The top layer with the dissolved carotenoids was collected, while the bottom layer was mixed with 2 ml of mixed solvent. The same procedure as before was followed until the bottom layer became colorless. The top n‐hexane layer was added to the previous one and the absorbance was measured at 450 nm using a spectrophotometer (UV‐1100, meipuda instrument, China). Pure n‐hexane was used as a reference. Total carotenoids content was calculated according to McBeth's formula as (Lin et al., 2011; Yuan et al., 2008).

$$\text{Carotenoids}\left(\text{mg}/100\;g\right)=1000\times A\times V/\left(E_{1\;\text{cm}}^{1\%}\times m\right).$$

where, absorbance (*A*) was determined by a spectrophotometer at 450 nm, *V* is the total volume of the solution (mL) and E_1cm_^1%^ is 1% of the average extinction coefficient value of carotenoids in n‐hexane = 2500, and *m* is the weight of the sample (g). The carotenoids concentration of digesta was determined by the same method. The bioaccessibility was calculated using the following equation:

$$\text{Bioaccessibility}\left(\%\right)=100\times C_{\text{micelle}}/C_{\text{digesta}}$$

where, *C*_micelle_ and *C*_digesta_ are the concentrations of carotenoids in the micelle fraction and in the overall sample (raw digesta) after the pH‐stat experiment, respectively.

### 2.7 Statistical analysis

All measurements were performed in triplicate and were presented as means and standard deviations.

## 3 RESULTS AND DISCUSSION

### 3.1 Influence of the mixed surfactants ratio on the formation of nanoemulsions

**FIGURE 1:**
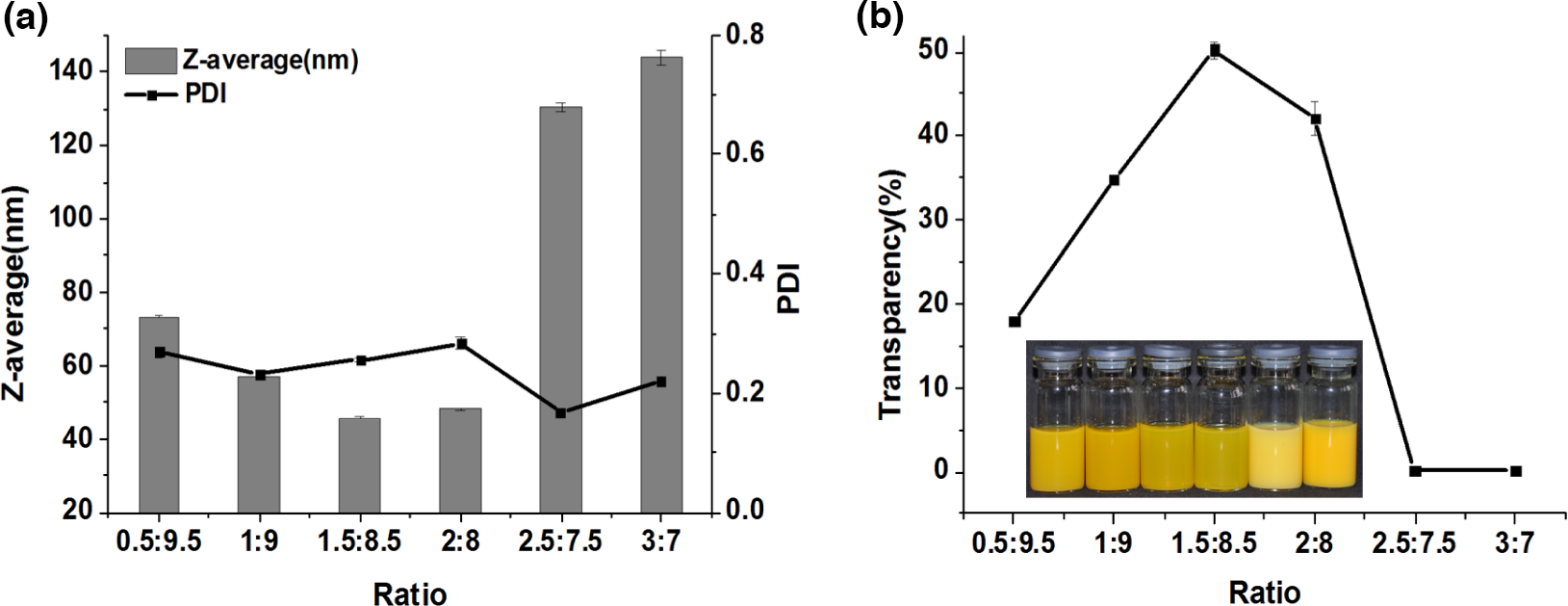
Effect of Span 80‐to‐Tween 80 ratio on (a) Z‐average, polydispersity index (PDI) and (b) transparency of nanoemulsions. Nanoemulsions were prepared using 5 wt% oil, surfactants‐to‐oil ratio (SOR) = 2, and stirring speed at 1000 rpm (revolutions per minute)

It is widely known that hydrophilic–lipophilic balance (HLB) value of the nonionic surfactants plays an important role in emulsions stability. Generally, surfactants with HLB values >10 are primarily hydrophilic to form oil‐in‐water (O/W) emulsions. In order to find the appropriate mixtures of hydrophilic emulsifiers, the influence of Span 80 and Tween 80 mixed mass ratio on the emulsions droplet size and transparency was studied (Figure 1). Initially, the droplet size decreased with the increasing ratio (Figure 1a). Droplet size reached the minimum at the ratio of 1.5:8.5, at which the HLB value was about 13.4. Then, the droplet size increased rapidly, with the increasing ratio indicating the surfactant compositions had significant influence on the droplet size. Although, the HLB of all the used mixed surfactants was >10. The result showed that nanoemulsions with the smallest droplet size were produced when the ratio of Span 80:Tween 80 was 1.5:8.5. It may be the mixed surfactants can well coat on the oil–water boundary. Previous study showed the size of the droplets produced depended strongly on the surfactant type. An optimum surfactant's molecular geometry will promote the spontaneous formation of small droplets at the oil–water boundary using low‐energy methods (Guttoff et al., 2015). PDI values varied from 0.17 to 0.28 at all the ratios, which indicated the ratio of mixed surfactants had a slight impact on PDI. Figure 1b displays that the transparency of samples was higher at smaller particle size, and vice versa. The small droplet size compared with the wavelength of light means they tend to be transparent. Indeed, the transparency of nanoemulsions formed using these methods changed with the increasing droplet size, which was similar to the results reported in other studies (Mayer et al., 2013; Saberi et al., 2013).

### 3.2 Influence of organic phase content and stirring speed on the formation of nanoemulsions

**FIGURE 2:**
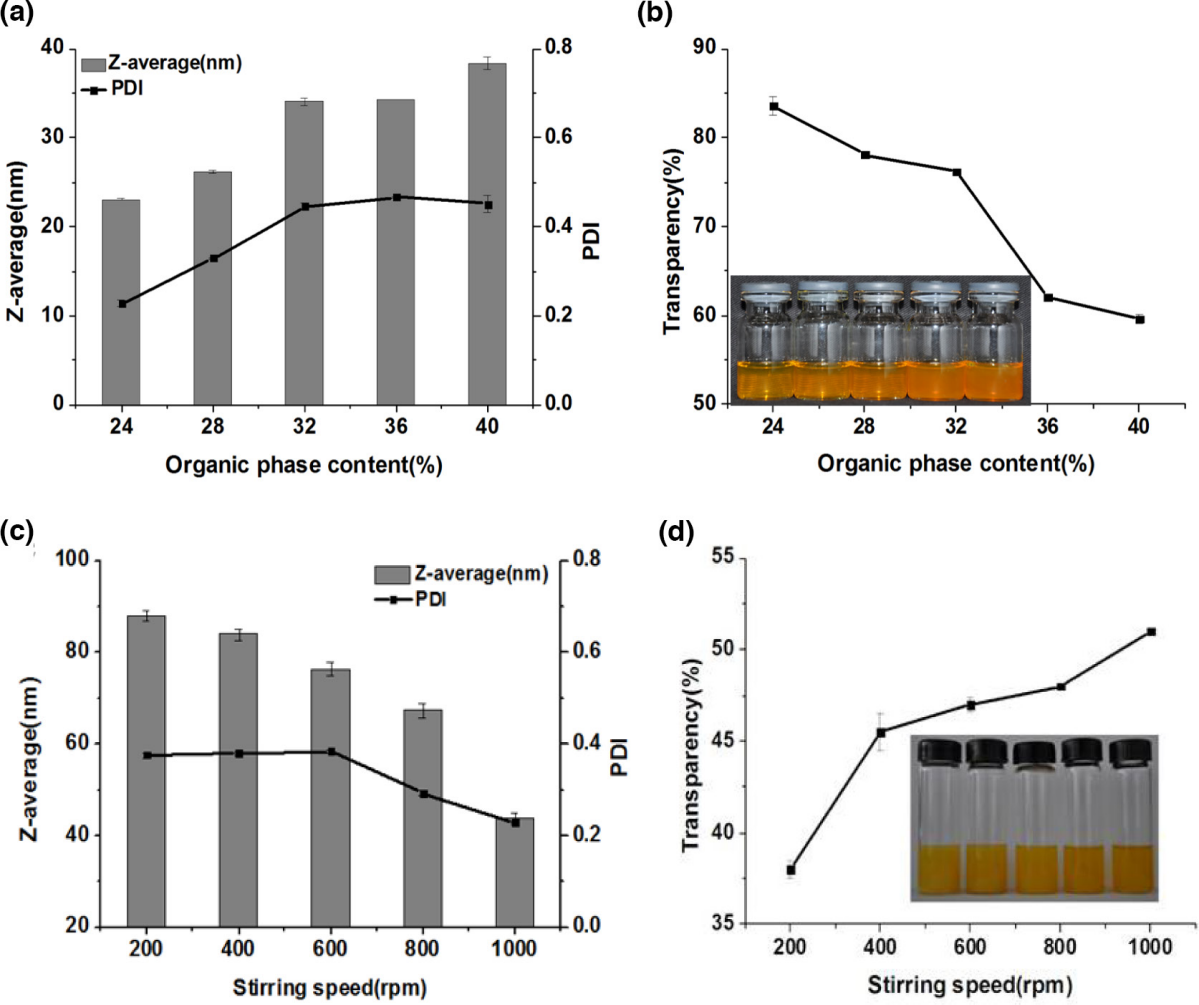
Effect of organic phase content (a, b) and stirring speed (c, d) on Z‐average, polydispersity index (PDI) and transparency of nanoemulsions. Nanoemulsions were prepared using (a, b) Span 80‐to‐Tween 80 ratio 1.5:8.5, surfactants‐to‐oil ratio (SOR)=3, and stirring speed at 1000 rpm (revolutions per speed); (c, d) 5 wt% oil, Span 80‐to‐Tween 80 ratio is 1.5:8.5, and SOR = 2

The impact of organic phase content on the droplet size of nanoemulsions was investigated (Figure 2). The droplet sizes of nanoemulsions slightly increased from 23 to 38 nm, with the organic phase content increasing (Figure 2a). Because when SOR was kept constant at 3, the water content decreased with the increase of organic phase content in the system and led to the system viscosity increase. The high viscosity does not favor the magnetic stirring and hinders the diffusion of organic phase to water phase during emulsification. Though the increase of droplet size was slight when the organic phase content was between 24% and 40%, the PDI obviously increased from 0.23 to 0.47. The transparency has decreased from 83.6% to 59.6%, with the droplet size increasing (Figure 2b).

The spontaneous emulsification method does not need any special homogenization equipment, but it requires some stirring during the titration of the organic phase into the aqueous phase. When the organic phases were added dropwise into aqueous phase, the diffusion velocity of water‐miscible components into the aqueous phase was slow and the droplet size was relatively big if there was no external stir. The intense disruptive forces are required for the rapid movement of the water‐miscible components, breaking droplets up and forming fine emulsions. Thus, stirring speed would affect the droplet size when emulsions were prepared by the spontaneous emulsification method. The droplet size decreased gradually with the increasing of stirring speed (Figure 2c). Nanoemulsions produced at 1000 rpm had the smallest droplet diameters (about 45 nm) (Figure 2c). Comparatively, the droplet size prepared at 200 rpm was about 90 nm, which is in agreement with other studies (Saberi et al., 2013). The PDI of nanoemulsions was from 0.229 to 0.383 at all the stirring speeds (Figure 2c). The transparency of nanoemulsions increased with the increasing stirring speed, and the highest transparency was obtained at 1000 rpm (Figure 2d).

### 3.3 Influence of SOR on the formation of nanoemulsions

**FIGURE 3:**
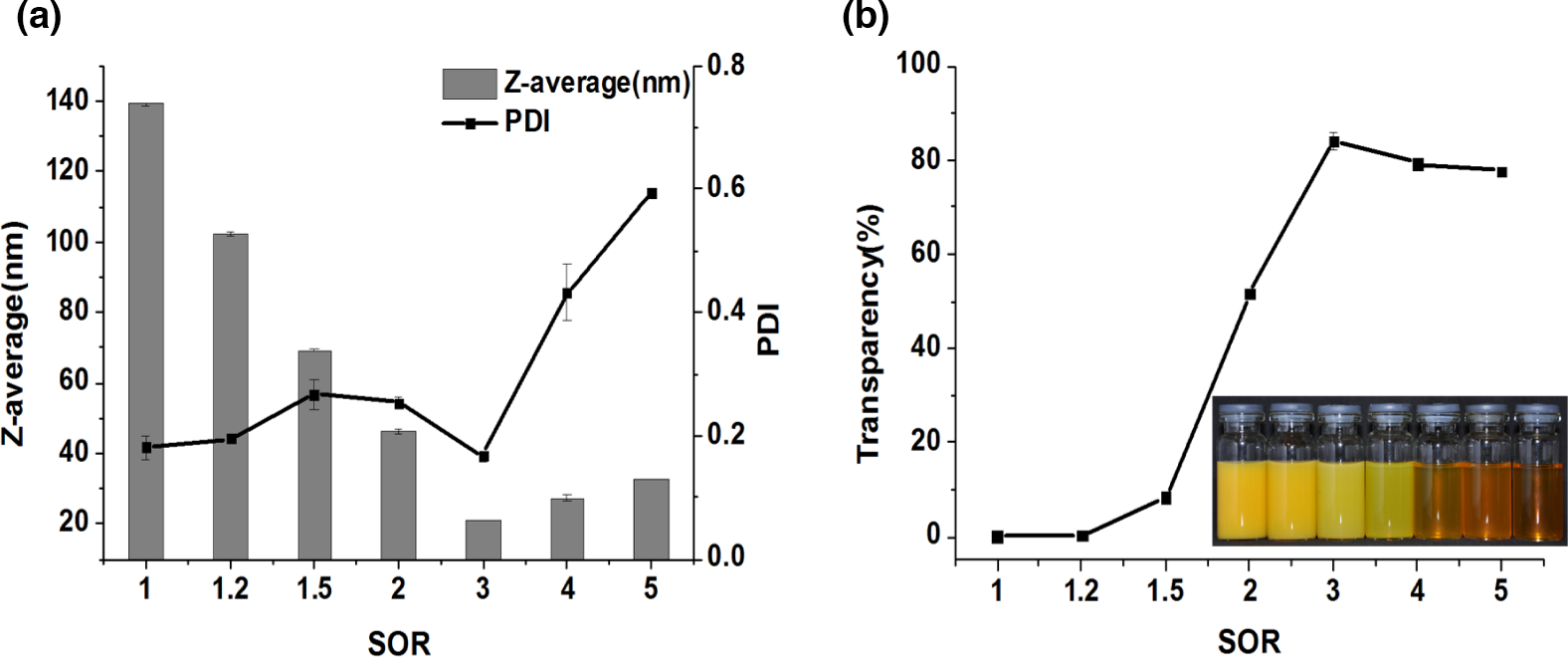
Effect of surfactants‐to‐oil ratio (SOR) on (a) Z‐average, polydispersity index (PDI) and (b) transparency of nanoemulsions. Nanoemulsions were prepared using 5 wt% oil, Span 80‐to‐Tween 80 ratio is 1.5:8.5, and stirring speed at 1000 rpm (revolutions per minute)

The influence of SOR on the formation of carotenoids nanoemulsions was investigated in this series of experiments. In these experiments, the total amount of MCT in the systems was kept constant (5 wt%), while the amount of surfactant was varied (5%–25%) with the remainder being buffer solution (90%–70%). The effects of SOR on the emulsions were investigated and the results are displayed in Figure 3. Increasing the SOR from 1 to 3 resulted in a significant decrease in the droplet size. A smaller droplet size means greater surface area, which requires more emulsifiers to cover. Furthermore, the increased adsorption of surfactant molecules to the oil–water interface leads to a decrease in the interfacial tension, which facilitates the formation of smaller droplets (Lamaallam, 2005). Other researchers have reported similar results that higher surfactant concentrations have produced smaller droplet sizes (Komaiko & Mcclements, 2014). The smallest droplets (about 20 nm) were formed at the SOR of 3, and the system was very transparent (Figure 3b). As SOR was higher than 3, the droplet size had a slight increase in trends, but the PDI increased dramatically from 0.17 to 0.59 (Figure 3a). It has been postulated that the droplet size increases above a certain surfactant level due to the formation of a highly viscous liquid crystalline phase, which makes spontaneous breakup of the oil–water interface more difficult (Mayer et al., 2013; Wang et al., 2009).

### 3.4 Size and surface charge of emulsions after digestion

**FIGURE 4:**
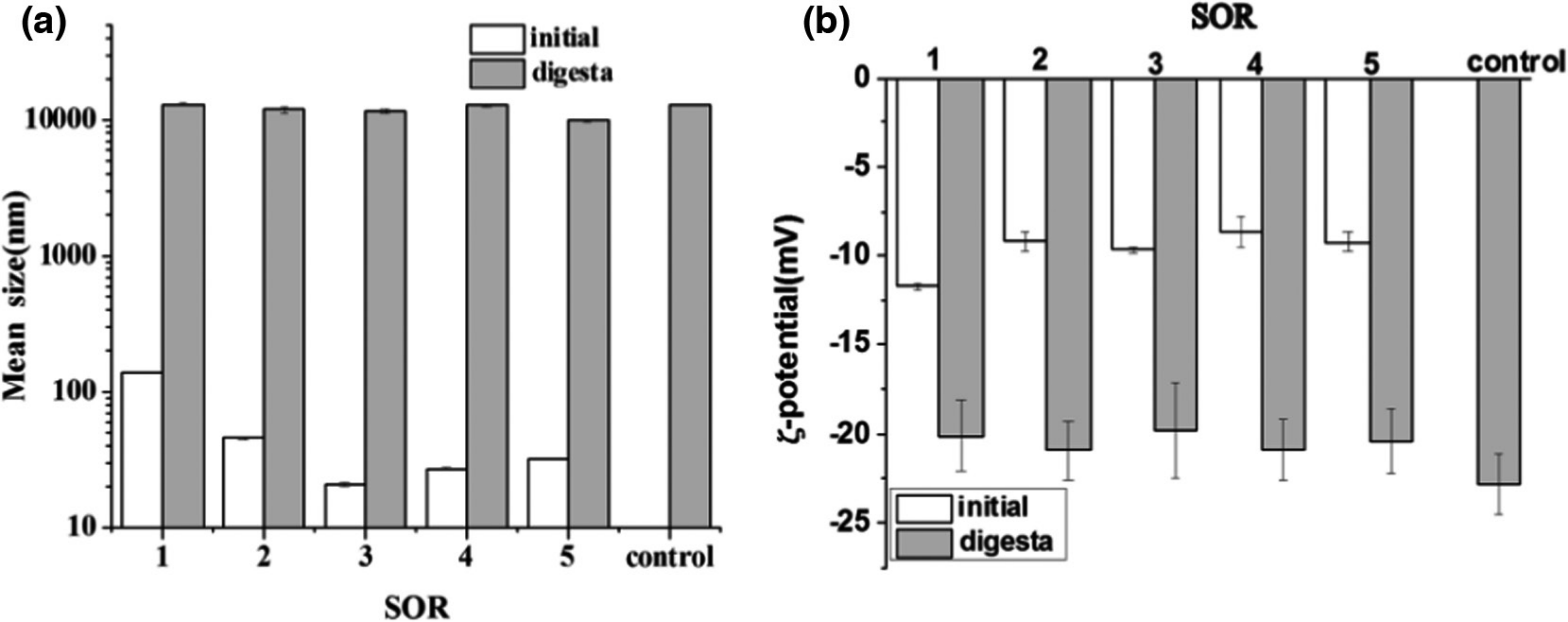
Mean particle size (a) and ζ‐potential (b) of nanoemulsions fabricated with different surfactant‐to‐oil ratios (SORs) before and after in vitro digestion

The mean particle size at all SORs increased appreciably from about 20–140 nm to about 10 μm, and had no obvious difference after in vitro digestion (Figure 4a). The results indicated that there was a significant reduction in the physical stability of the emulsions after in vitro digestion. The increase in mean particle size after simulated digestion may be due to flocculation and/or coalescence phenomena depending on the nature of the system (Mayer et al., 2013; Wang et al., 2009). On one hand, the highly surface‐active bile salts may have displaced some or all of the nonionic surfactants Tween 80 and Span 80 from the oil droplet surfaces. On the other hand, lipase could be adsorbed onto oil droplet surfaces and convert triglyceride into free fatty acids (FFAs) and monoacylglycerols (MAGs). The removal of FFAs’ and MAGs’ digestion products from fat droplets would lead to a reduction in the size. But droplet coalescence, caused by lipid digestion, would lead to an increase in the size.

The surface charges of emulsions droplets before and after digestion were also measured to provide some information about changes in interfacial properties (Figure 4b). The negative charge of the initial nanoemulsions is about −10 mV. The magnitude of the negative charge on the particles in the digesta increased appreciably, with the values ranging from about −10 to −20 mV. When lipid droplets were exposed to simulated intestinal conditions that contained bile salts, which are anionic surface‐active substances, the bile salts may displace the nonionic surfactants molecules from their surfaces and generate a negative charge (Mcclements & Hang, 2012; Qian et al., 2012). In addition, the hydrolysis of neutral triglycerides by pancreatic lipase at the surface will lead to the release of anionic FFAs. Any of these molecules that remained at the droplet surfaces will also produce a negative charge. Different SORs do not cause much difference in the electrical characteristics of the particles in the digesta. It means that some or all the surfactants on the droplet surface have been replaced by bile salts or other molecules.

### 3.5 Influence of SOR on lipids digestibility

**FIGURE 5:**
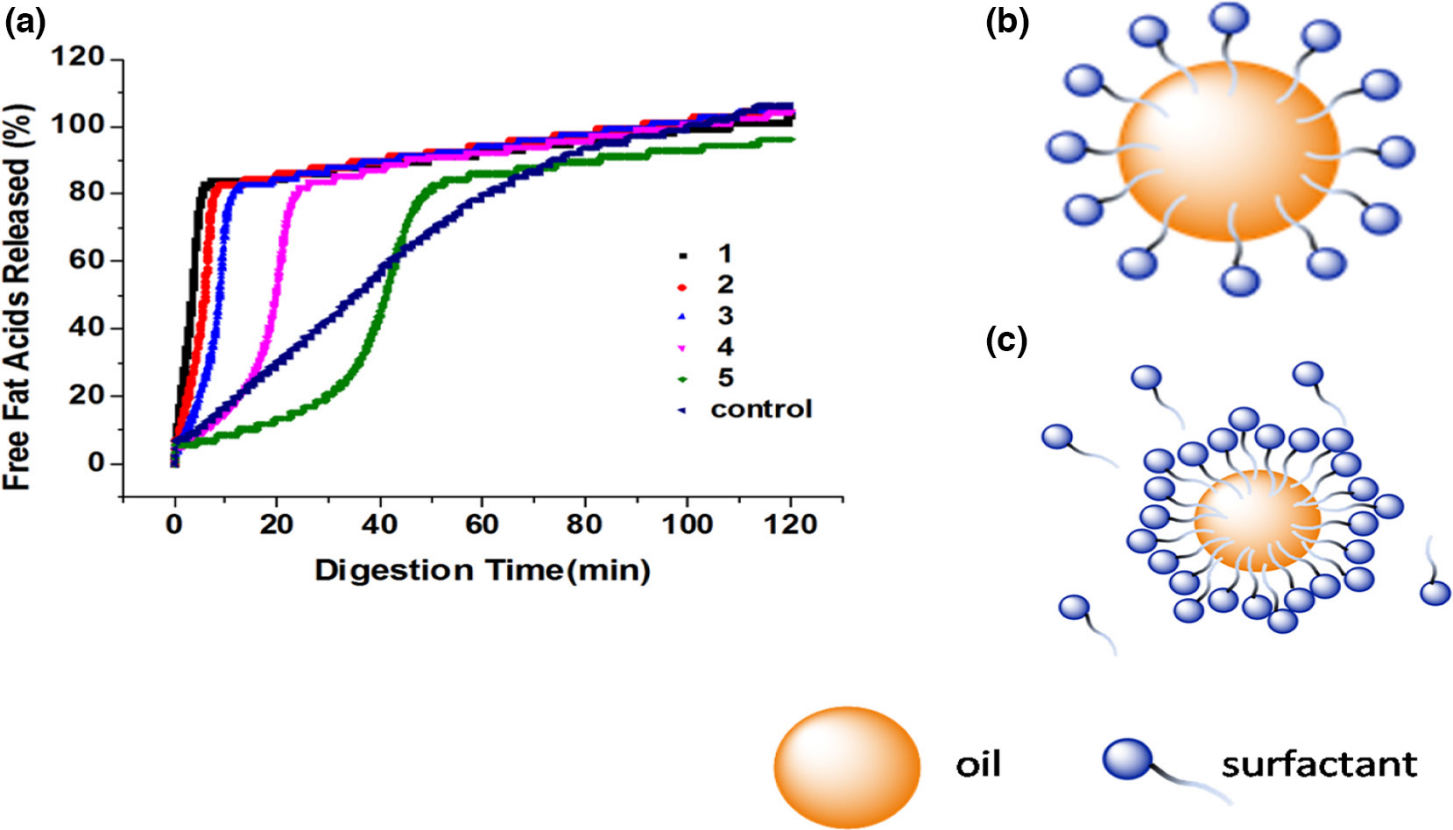
Influence of surfactant‐to‐oil ratio (SOR) on lipids digestion and scheme of the emulsion droplets’ interface

The influence of SOR on the rate and extent of triglyceride digestion in the nanoemulsions using the pH‐stat method was investigated (Figure 5). The initial rate of lipid digestion decreased as the SOR increased from 1 to 5. At low SOR (≤3), the rate of lipid digestion was very fast and the amount of released FFA increased steeply during the first 10 min after adding pancreatic lipase into the digestion medium. Most of the MCT within the droplets was digested after 10 min (>80% FFA released), and then the digestion rate exhibited a slower increase as the time extended. At high SOR (>3), the release rate of FFAs was much slower. The released FFAs from nanoemulsions formed at SOR 5 reached 80% after 50 min digestion, and reached 96% after 2 hr. The difference of digestion rate and extent between prepared emulsions with different SORs may have resulted from the different contents of surfactants. Superfluous surfactants could prevent bile salts and/or lipase from contacting lipid in the emulsions. The small molecule surfactants are surface‐active, so that any free nonionic surfactants in the continuous phase may interact with the lipase or compete with it in the oil–water interface, and decrease the lipase adsorption and activity. The carotenoids dissolved in bulk MCT referred to as the control sample, which showed a much slower digestion rate. The results suggested that the lipid digestion was an interfacial phenomenon (Mcclements & Yan, 2010). The bulk lipids had a smaller surface area, which delayed the interaction with lipase.

### 3.6 Effect of SOR on the bioaccessibility of carotenoids

**FIGURE 6:**
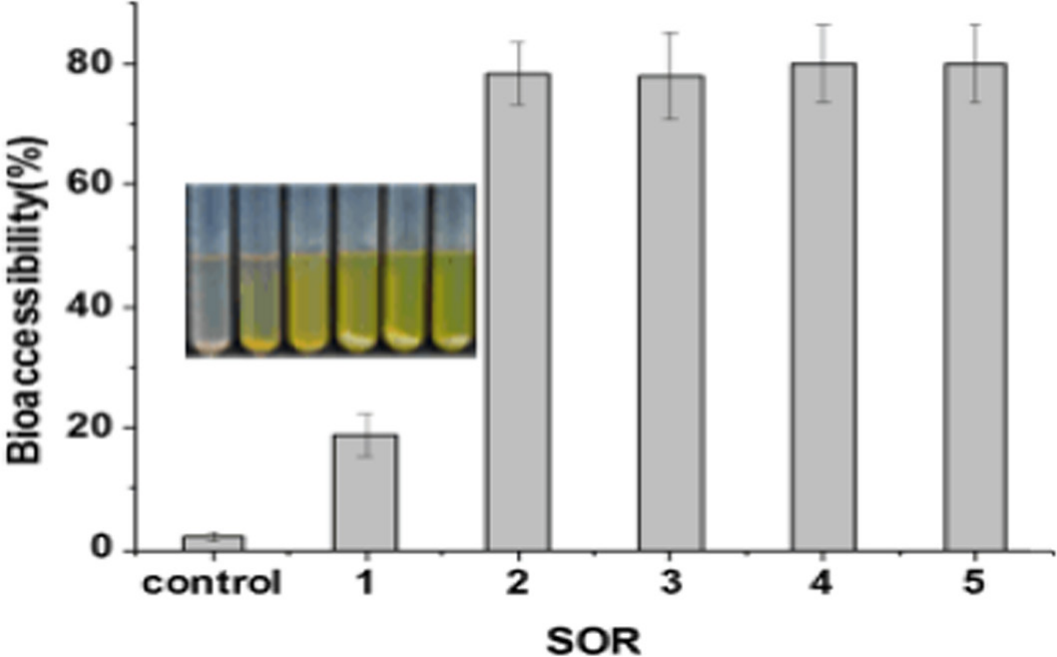
Effect of surfactant‐to‐oil ratio (SOR) on the bioaccessibility of carotenoids incorporated in nanoemulsions

The nanoemulsion systems showed a greater bioaccessibility than the bulk lipid sample (Figure 6). The bioaccessibility of carotenoids dispersed in bulk lipid was only 2.4 ± 0.3%. Comparatively, the emulsions system showed a bioaccessibility of about 80% at SOR = 2–5. This phenomenon was related to the micellization process during digestion. When the bile salts were adsorbed onto the droplet surfaces and displaced any existing emulsifier molecules, the micelles were formed gradually with the carotenoids and products of digestion e.g. FFAs and MAGs. The bioaccessibility values at SOR = 1 were significantly different from those of other emulsions, which may be due to the relatively big droplet size (140 nm). Generally smaller droplet size, i.e. higher surface area, was available for pancreatic lipases to attach more easily, resulting in an increased transfer of nutraceuticals to micelles (Yi et al., 2014). Previous study also showed that the initial droplet size will affect the transfer efficiency of carotene (Pan et al., 2012). The results suggested that SOR will affect both droplet size and micelle formation. When SOR increased from 2 to 5, the bioaccessibility of carotenoids was similar owing to their small droplet size (20–60 nm). However, some emulsifiers may be able to participate in the formation of micelles. Therefore, the actual metabolic pathway that carotenoids in nanoemulsions undergo in vivo digestion should be investigated for reasonable design of the nanoemulsion‐based delivery system.

## 4 CONCLUSION

Carotenoids from *Lycium barbarum* can be successfully incorporated into food‐grade nanoemulsions with nonionic surfactants by spontaneous emulsification. The ratio of mixed surfactants had a major effect on droplet size, with the smallest droplets being formed at Span 80:Tween 80 = 1.5:8.5 (W/W). SOR also had an appreciable impact on droplet size. By optimizing system composition, carotenoids‐loaded unimodal nanoemulsions with small mean droplet diameters (d < 50 nm) could be formed. Meanwhile, SOR has significant influence on the oil digestion rate. The initial rate of lipid digestion decreased as the SOR increased from 1 to 5, and the bioaccessibility could reach about 80% at SOR = 2–5. Overall, this study provides valuable information for designing nutraceutical delivery systems, which can highly enhance hydrophilicity and bioaccessibility of lipophilic compounds.

Ideally, people would like to be able to form small droplets using the lowest amount of surfactant possible for economic, taste, and safety reasons. The result showed that nanoemulsions with small droplet size and high bioaccessibility of carotenoids could be formed at SOR ≥2 (surfactant concentration ≥10%). The value is much higher than that of fabricated nanoemulsions using high‐energy methods. Self‐emulsifying methods do not need expensive equipment and much energy, and only use simple stirring. Moreover, it is easy to fabricate fine nanoemulsions. The nanoemulsions can be diluted a lot of times and used in actual application to make it suitable for human consumption. Furthermore, in vivo studies would be useful to better understand the various physicochemical mechanisms that determine triglyceride digestion and the release of bioactive compounds from nanoemulsions system.

## CONFLICTS OF INTEREST

The authors declare that they do not have any conflicts of interest.

## Data Availability

The data that support the findings of this study are available from the corresponding author upon reasonable request.
